# 7-Bromo-2-(4-fluoro­phen­yl)-1-(methyl­sulfin­yl)naphtho[2,1-*b*]furan

**DOI:** 10.1107/S1600536810011645

**Published:** 2010-04-02

**Authors:** Hong Dae Choi, Pil Ja Seo, Byeng Wha Son, Uk Lee

**Affiliations:** a Department of Chemistry, Dongeui University, San 24 Kaya-dong Busanjin-gu, Busan 614-714, Republic of Korea; b Department of Chemistry, Pukyong National University, 599-1 Daeyeon 3-dong, Nam-gu, Busan 608-737, Republic of Korea

## Abstract

In the title compound, C_19_H_12_BrFO_2_S, the O atom and the methyl group of the methyl­sulfinyl substituent lie on opposite sides of the plane through the naphthofuran fragment. The 4-fluoro­phenyl ring is rotated out of the naphthofuran plane, making a dihedral angle of 41.65 (7)°. In the crystal, mol­ecules are linked by weak inter­molecular C—H⋯O and C—H⋯π inter­actions, and a short Br⋯F contact [3.046 (2) Å] occurs. The O atom of the sulfinyl group is disordered over two positions, with refined site-occupancy factors of 0.912 (4) and 0.088 (4).

## Related literature

For the crystal structures of similar 7-bromo-2-phenyl­naphtho[2,1-*b*]furan derivatives, see: Choi *et al.* (2006 ▶, 2009 ▶). For the biological activity of naphthofuran compounds, see: Einhorn *et al.* (1984 ▶); Hranjec *et al.* (2003 ▶); Mahadevan & Vaidya (2003 ▶).
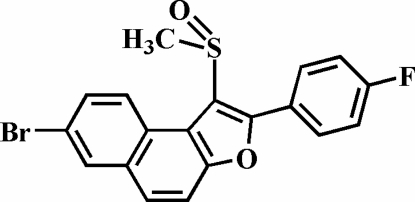


## Experimental

### 

#### Crystal data

C_19_H_12_BrFO_2_S
*M* *_r_* = 403.26
Monoclinic, 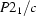

*a* = 6.0155 (2) Å
*b* = 22.7143 (6) Å
*c* = 11.4364 (3) Å
β = 91.716 (1)°
*V* = 1561.94 (8) Å^3^
*Z* = 4
Mo *K*α radiation
μ = 2.79 mm^−1^
*T* = 173 K
0.31 × 0.28 × 0.16 mm

#### Data collection

Bruker SMART APEXII CCD diffractometer
Absorption correction: multi-scan (*SADABS*; Bruker, 2009 ▶) *T*_min_ = 0.515, *T*_max_ = 0.746
14194 measured reflections
3547 independent reflections
3086 reflections with *I* > 2σ(*I*)
*R*_int_ = 0.031

#### Refinement

*R*[*F* ^2^ > 2σ(*F* ^2^)] = 0.032
*wR*(*F* ^2^) = 0.078
*S* = 1.09
3547 reflections
228 parameters
1 restraint
H-atom parameters constrained
Δρ_max_ = 0.55 e Å^−3^
Δρ_min_ = −0.66 e Å^−3^

### 

Data collection: *APEX2* (Bruker, 2009 ▶); cell refinement: *SAINT* (Bruker, 2009 ▶); data reduction: *SAINT*; program(s) used to solve structure: *SHELXS97* (Sheldrick, 2008 ▶); program(s) used to refine structure: *SHELXL97* (Sheldrick, 2008 ▶); molecular graphics: *ORTEP-3* (Farrugia, 1997 ▶) and *DIAMOND* (Brandenburg, 1998 ▶); software used to prepare material for publication: *SHELXL97*.

## Supplementary Material

Crystal structure: contains datablocks global, I. DOI: 10.1107/S1600536810011645/zl2274sup1.cif

Structure factors: contains datablocks I. DOI: 10.1107/S1600536810011645/zl2274Isup2.hkl

Additional supplementary materials:  crystallographic information; 3D view; checkCIF report

## Figures and Tables

**Table 1:** Hydrogen-bond geometry (Å, °) *Cg* is the centroid of the C2/C3/C8/C9/C10/C11 ring.

*D*—H⋯*A*	*D*—H	H⋯*A*	*D*⋯*A*	*D*—H⋯*A*
C14—H14⋯O2*A*^i^	0.93	2.53	3.235 (3)	133
C18—H18⋯*Cg*^ii^	0.93	2.65	3.347 (3)	132

Symmetry codes: (i) 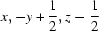; (ii) 
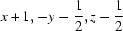
.

**Fig. 1.**
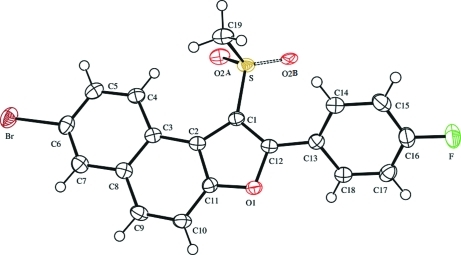
The molecular structure of the title compound with the atom numbering scheme. Displacement ellipsoids are drawn at the 50% probability level. H atoms are presented as a small spheres of arbitrary radius. The bond towards the minor occupied oxygen atom is shown in a dashed mode.

**Fig. 2.**
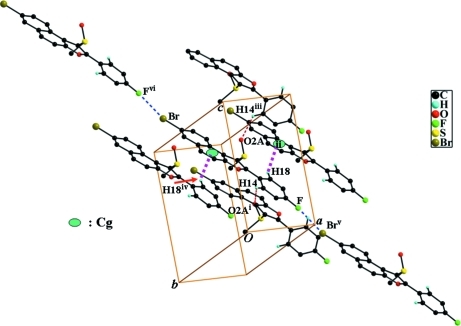
C–H···O, C–H···π and Br···F interactions (dotted lines) in the crystal structure of the title compound. Cg denotes the ring centroid. The disordered component of the oxygen of sulfinyl group, part B, has been omitted for clairty. [Symmetry codes: (i) x, - y + 1/2, z - 1/2; (ii) x + 1, y, z ; (iii) x, - y + 1/2, z + 1/2; (iv) x - 1, y, z; (v) x + 2, y, z - 1; (vi) x - 2, y, z + 1.]

## supplementary crystallographic information

### Comment

Many compounds containing naphthofuran moieties show potent biological activities such as antibacterial (Einhorn *et al.*, 1984), antitumor (Hranjec *et al.*, 2003) and anthelmintic (Mahadevan & Vaidya, 2003) properties. As a part of our continuing studies of the effect of side chain substituents on the solid state structures of 7-bromo-2-phenylnaphtho[2,1-b]furan analogues (Choi *et al.*, 2006, 2009), we report the crystal structure of the title compound (Fig. 1).

The naphthofuran unit is essentially planar, with a mean deviation of 0.040 (2) Å from the least-squares plane defined by the thirteen constituent atoms. The oxygen of the sulfinyl group is disordered over two positions with site-occupancy factors of 0.912 (4) (for O atom labeled A) and 0.088 (4) (for O atom labeled B). The dihedral angle formed by the naphthofuran plane and the 4-fluorophenyl ring is 41.65 (7)°. The crystal packing (Fig. 2) is stabilized by intermolecular C–H···O hydrogen bonds between the 4-fluorophenyl H atom H14 and the oxygen O2A^i^ of the S═O unit. The molecular packing (Fig. 2) is further stabilized by intermolecular C–H···π interactions between the 4-fluorophenyl H atom H18 and the centroid Cg^ii^ of the central benzene ring of an adjacent naphthofuran system (see Table 1 for numerical values and symmetry operators; Cg is the centroid of the atoms C2/C3/C8/C9/C10/C11 of the benzene ring). Furthermore, a short Br···F^iv^ contact (Fig. 2) [3.046 (2) Å] provides additional stabilization.

### Experimental

77% 3-Chloroperoxybenzoic acid (202 mg, 0.9 mmol) was added in small portions to a stirred solution of 7-bromo-2-(4-fluorophenyl)-1-(methylsulfanyl)naphtho[2,1-b]furan (310 mg, 0.8 mmol) in dichloromethane (30 mL) at 273 K. After being stirred at room temperature for 5h, the mixture was washed with saturated sodium bicarbonate solution and the organic layer was separated, dried over magnesium sulfate, filtered and concentrated at reduced pressure. The residue was purified by column chromatography (hexane-ethyl acetate, 1:1 v/v) to afford the title compound as a colorless solid [yield 76%, m.p. 501-502 K; *R*_f_ = 0.66 (hexane-ethyl acetate, 1:1 v/v)]. Single crystals suitable for X-ray diffraction were prepared by slow evaporation of a solution of the title compound in chloroform at room temperature.

### Refinement

All H atoms were geometrically positioned and refined using a riding model, with C–H = 0.95 Å for aryl and 0.98 Å for methyl H atoms. *U*_iso_(H) = 1.2*U*_eq_(C) for aryl and 1.5*U*_eq_(C) for methyl H atoms. The S═O distances (A & B) were restrained to be the same within a standard deviation of 0.002 Å using SADI command as defined in SHELXTL (Sheldrick, 2008).

### Crystal data

**Table:**

C_19_H_12_BrFO_2_S	*F*(000) = 808
*M**_r_* = 403.26	*D*_x_ = 1.715 Mg m^−^^3^
Monoclinic, *P*2_1_/*c*	Mo *K*α radiation, λ = 0.71073 Å
Hall symbol: -P 2ybc	Cell parameters from 6681 reflections
*a* = 6.0155 (2) Å	θ = 2.5–27.3°
*b* = 22.7143 (6) Å	µ = 2.79 mm^−^^1^
*c* = 11.4364 (3) Å	*T* = 173 K
β = 91.716 (1)°	Block, colourless
*V* = 1561.94 (8) Å^3^	0.31 × 0.28 × 0.16 mm
*Z* = 4	

### Data collection

**Table:**

Bruker SMART APEXII CCD diffractometer	3547 independent reflections
Radiation source: Rotating Anode	3086 reflections with *I* > 2σ(*I*)
Bruker HELIOS graded multilayer optics	*R*_int_ = 0.031
Detector resolution: 10.0 pixels mm^-1^	θ_max_ = 27.4°, θ_min_ = 1.8°
φ and ω scans	*h* = −7→7
Absorption correction: multi-scan (*SADABS*; Bruker, 2009)	*k* = −29→28
*T*_min_ = 0.515, *T*_max_ = 0.746	*l* = −14→14
14194 measured reflections	

### Refinement

**Table:**

Refinement on *F*^2^	Primary atom site location: structure-invariant direct methods
Least-squares matrix: full	Secondary atom site location: difference Fourier map
*R*[*F*^2^ > 2σ(*F*^2^)] = 0.032	Hydrogen site location: difference Fourier map
*wR*(*F*^2^) = 0.078	H-atom parameters constrained
*S* = 1.09	*w* = 1/[σ^2^(*F*_o_^2^) + (0.0285*P*)^2^ + 1.3174*P*] where *P* = (*F*_o_^2^ + 2*F*_c_^2^)/3
3547 reflections	(Δ/σ)_max_ < 0.001
228 parameters	Δρ_max_ = 0.55 e Å^−^^3^
1 restraint	Δρ_min_ = −0.66 e Å^−^^3^

### Special details

**Table:**

Geometry. All esds (except the esd in the dihedral angle between two l.s. planes) are estimated using the full covariance matrix. The cell esds are taken into account individually in the estimation of esds in distances, angles and torsion angles; correlations between esds in cell parameters are only used when they are defined by crystal symmetry. An approximate (isotropic) treatment of cell esds is used for estimating esds involving l.s. planes.
Refinement. Refinement of F^2^ against ALL reflections. The weighted R-factor wR and goodness of fit S are based on F^2^, conventional R-factors R are based on F, with F set to zero for negative F^2^. The threshold expression of F^2^ > 2sigma(F^2^) is used only for calculating R-factors(gt) etc. and is not relevant to the choice of reflections for refinement. R-factors based on F^2^ are statistically about twice as large as those based on F, and R- factors based on ALL data will be even larger.

### Fractional atomic coordinates and isotropic or equivalent isotropic displacement parameters (Å^2^)

**Table:**

	*x*	*y*	*z*	*U*_iso_*/*U*_eq_	Occ. (<1)
Br	−0.48150 (5)	0.382579 (12)	1.04709 (2)	0.03916 (10)	
S	0.47791 (10)	0.28043 (2)	0.66043 (5)	0.02628 (13)	
F	1.2163 (3)	0.36776 (7)	0.25403 (14)	0.0483 (4)	
O1	0.5560 (3)	0.45183 (6)	0.62399 (13)	0.0243 (3)	
O2A	0.4525 (3)	0.25792 (7)	0.78045 (15)	0.0328 (5)	0.912 (4)
O2B	0.6982 (13)	0.2602 (6)	0.6238 (14)	0.024 (5)	0.088 (4)
C1	0.4554 (4)	0.35832 (9)	0.66166 (18)	0.0218 (4)	
C2	0.3135 (4)	0.39689 (9)	0.72727 (18)	0.0218 (4)	
C3	0.1281 (4)	0.39074 (9)	0.80143 (17)	0.0224 (4)	
C4	0.0413 (4)	0.33627 (10)	0.84002 (19)	0.0267 (5)	
H4	0.1074	0.3013	0.8168	0.032*	
C5	−0.1380 (4)	0.33420 (10)	0.9107 (2)	0.0302 (5)	
H5	−0.1931	0.2982	0.9353	0.036*	
C6	−0.2378 (4)	0.38667 (10)	0.94578 (19)	0.0284 (5)	
C7	−0.1620 (4)	0.44040 (10)	0.91127 (18)	0.0281 (5)	
H7	−0.2329	0.4746	0.9349	0.034*	
C8	0.0242 (4)	0.44381 (9)	0.83966 (18)	0.0245 (4)	
C9	0.1093 (4)	0.49995 (9)	0.80711 (18)	0.0272 (5)	
H9	0.0393	0.5338	0.8332	0.033*	
C10	0.2894 (4)	0.50541 (9)	0.73923 (18)	0.0265 (5)	
H10	0.3465	0.5420	0.7197	0.032*	
C11	0.3846 (4)	0.45309 (9)	0.70009 (17)	0.0226 (4)	
C12	0.5951 (4)	0.39335 (9)	0.60055 (18)	0.0222 (4)	
C13	0.7632 (4)	0.38339 (9)	0.51270 (18)	0.0227 (4)	
C14	0.7334 (4)	0.34109 (10)	0.42469 (19)	0.0275 (5)	
H14	0.6091	0.3167	0.4245	0.033*	
C15	0.8873 (4)	0.33521 (10)	0.3378 (2)	0.0314 (5)	
H15	0.8682	0.3072	0.2791	0.038*	
C16	1.0688 (4)	0.37188 (10)	0.3406 (2)	0.0322 (5)	
C17	1.1043 (4)	0.41416 (10)	0.4261 (2)	0.0295 (5)	
H17	1.2296	0.4382	0.4256	0.035*	
C18	0.9504 (4)	0.41994 (9)	0.51190 (19)	0.0252 (4)	
H18	0.9708	0.4483	0.5698	0.030*	
C19	0.2271 (4)	0.26380 (10)	0.5786 (2)	0.0328 (5)	
H19A	0.2018	0.2221	0.5796	0.049*	
H19B	0.2409	0.2768	0.4993	0.049*	
H19C	0.1042	0.2836	0.6131	0.049*	

### Atomic displacement parameters (Å^2^)

**Table:**

	*U*^11^	*U*^22^	*U*^33^	*U*^12^	*U*^13^	*U*^23^
Br	0.03555 (15)	0.05091 (17)	0.03161 (14)	−0.01198 (11)	0.01077 (10)	−0.00502 (11)
S	0.0273 (3)	0.0176 (2)	0.0338 (3)	0.0016 (2)	−0.0020 (2)	0.0026 (2)
F	0.0514 (10)	0.0486 (9)	0.0464 (9)	0.0068 (8)	0.0279 (8)	−0.0001 (7)
O1	0.0293 (8)	0.0186 (7)	0.0251 (7)	−0.0030 (6)	0.0028 (6)	0.0004 (6)
O2A	0.0417 (12)	0.0238 (9)	0.0326 (10)	−0.0001 (8)	−0.0051 (8)	0.0102 (7)
O2B	0.036 (11)	0.010 (7)	0.025 (9)	−0.003 (7)	0.003 (7)	0.007 (6)
C1	0.0228 (11)	0.0192 (9)	0.0233 (10)	0.0001 (8)	−0.0023 (8)	0.0019 (8)
C2	0.0258 (11)	0.0199 (10)	0.0193 (9)	0.0000 (8)	−0.0033 (8)	0.0016 (7)
C3	0.0263 (11)	0.0229 (10)	0.0177 (9)	−0.0020 (8)	−0.0031 (8)	0.0008 (8)
C4	0.0294 (12)	0.0228 (10)	0.0277 (11)	−0.0014 (9)	−0.0008 (9)	0.0027 (8)
C5	0.0323 (13)	0.0297 (11)	0.0285 (11)	−0.0081 (10)	−0.0003 (9)	0.0042 (9)
C6	0.0257 (12)	0.0389 (13)	0.0207 (10)	−0.0049 (10)	0.0013 (8)	0.0001 (9)
C7	0.0320 (13)	0.0304 (11)	0.0218 (10)	0.0015 (9)	0.0009 (9)	−0.0005 (9)
C8	0.0298 (12)	0.0257 (10)	0.0178 (9)	0.0006 (9)	−0.0012 (8)	0.0001 (8)
C9	0.0392 (14)	0.0205 (10)	0.0219 (10)	0.0034 (9)	0.0031 (9)	−0.0010 (8)
C10	0.0378 (13)	0.0184 (10)	0.0234 (10)	−0.0017 (9)	0.0014 (9)	0.0006 (8)
C11	0.0254 (11)	0.0227 (10)	0.0198 (10)	−0.0024 (8)	0.0004 (8)	0.0006 (8)
C12	0.0254 (11)	0.0187 (10)	0.0222 (10)	−0.0003 (8)	−0.0032 (8)	−0.0007 (7)
C13	0.0237 (11)	0.0220 (10)	0.0222 (10)	0.0012 (8)	−0.0008 (8)	0.0030 (8)
C14	0.0298 (12)	0.0241 (10)	0.0286 (11)	−0.0009 (9)	−0.0007 (9)	−0.0006 (9)
C15	0.0408 (14)	0.0267 (11)	0.0269 (11)	0.0049 (10)	0.0028 (10)	−0.0031 (9)
C16	0.0353 (14)	0.0326 (12)	0.0294 (12)	0.0094 (10)	0.0094 (10)	0.0058 (9)
C17	0.0255 (12)	0.0281 (11)	0.0349 (12)	−0.0001 (9)	0.0015 (9)	0.0082 (9)
C18	0.0271 (12)	0.0231 (10)	0.0250 (11)	0.0002 (9)	−0.0032 (9)	0.0011 (8)
C19	0.0353 (14)	0.0251 (11)	0.0375 (13)	−0.0045 (10)	−0.0070 (10)	−0.0012 (9)

### Geometric parameters (Å, °)

**Table:**

F—Br^i^	3.0457 (15)	C7—H7	0.9300
Br—C6	1.898 (2)	C8—C9	1.428 (3)
S—O2B	1.475 (3)	C9—C10	1.357 (3)
S—O2A	1.4769 (18)	C9—H9	0.9300
S—C1	1.775 (2)	C10—C11	1.399 (3)
S—C19	1.792 (2)	C10—H10	0.9300
F—C16	1.352 (3)	C12—C13	1.464 (3)
O1—C11	1.370 (3)	C13—C18	1.399 (3)
O1—C12	1.377 (2)	C13—C14	1.399 (3)
C1—C12	1.365 (3)	C14—C15	1.385 (3)
C1—C2	1.448 (3)	C14—H14	0.9300
C2—C11	1.384 (3)	C15—C16	1.373 (4)
C2—C3	1.428 (3)	C15—H15	0.9300
C3—C4	1.419 (3)	C16—C17	1.383 (3)
C3—C8	1.432 (3)	C17—C18	1.376 (3)
C4—C5	1.368 (3)	C17—H17	0.9300
C4—H4	0.9300	C18—H18	0.9300
C5—C6	1.398 (3)	C19—H19A	0.9600
C5—H5	0.9300	C19—H19B	0.9600
C6—C7	1.365 (3)	C19—H19C	0.9600
C7—C8	1.409 (3)		
			
C16—F—Br^i^	168.86 (14)	C8—C9—H9	119.0
O2B—S—O2A	106.0 (6)	C9—C10—C11	116.5 (2)
O2B—S—C1	112.5 (6)	C9—C10—H10	121.7
O2A—S—C1	109.13 (10)	C11—C10—H10	121.7
O2B—S—C19	122.4 (7)	O1—C11—C2	111.52 (18)
O2A—S—C19	107.67 (12)	O1—C11—C10	122.99 (19)
C1—S—C19	98.68 (11)	C2—C11—C10	125.4 (2)
C11—O1—C12	106.28 (16)	C1—C12—O1	110.56 (19)
C12—C1—C2	107.11 (18)	C1—C12—C13	135.23 (19)
C12—C1—S	121.91 (17)	O1—C12—C13	114.09 (17)
C2—C1—S	130.82 (16)	C18—C13—C14	119.3 (2)
C11—C2—C3	118.38 (19)	C18—C13—C12	119.03 (19)
C11—C2—C1	104.53 (18)	C14—C13—C12	121.5 (2)
C3—C2—C1	137.00 (19)	C15—C14—C13	120.5 (2)
C4—C3—C2	124.9 (2)	C15—C14—H14	119.7
C4—C3—C8	118.1 (2)	C13—C14—H14	119.7
C2—C3—C8	117.05 (18)	C16—C15—C14	118.3 (2)
C5—C4—C3	121.2 (2)	C16—C15—H15	120.9
C5—C4—H4	119.4	C14—C15—H15	120.9
C3—C4—H4	119.4	F—C16—C15	118.7 (2)
C4—C5—C6	119.5 (2)	F—C16—C17	118.4 (2)
C4—C5—H5	120.2	C15—C16—C17	122.9 (2)
C6—C5—H5	120.2	C18—C17—C16	118.5 (2)
C7—C6—C5	121.9 (2)	C18—C17—H17	120.7
C7—C6—Br	119.41 (18)	C16—C17—H17	120.7
C5—C6—Br	118.63 (17)	C17—C18—C13	120.5 (2)
C6—C7—C8	119.7 (2)	C17—C18—H18	119.8
C6—C7—H7	120.2	C13—C18—H18	119.8
C8—C7—H7	120.2	S—C19—H19A	109.5
C7—C8—C9	119.9 (2)	S—C19—H19B	109.5
C7—C8—C3	119.5 (2)	H19A—C19—H19B	109.5
C9—C8—C3	120.6 (2)	S—C19—H19C	109.5
C10—C9—C8	122.0 (2)	H19A—C19—H19C	109.5
C10—C9—H9	119.0	H19B—C19—H19C	109.5
			
O2B—S—C1—C12	19.7 (7)	C8—C9—C10—C11	1.6 (3)
O2A—S—C1—C12	137.02 (18)	C12—O1—C11—C2	0.6 (2)
C19—S—C1—C12	−110.77 (19)	C12—O1—C11—C10	−176.4 (2)
O2B—S—C1—C2	−155.0 (7)	C3—C2—C11—O1	−177.03 (17)
O2A—S—C1—C2	−37.8 (2)	C1—C2—C11—O1	0.1 (2)
C19—S—C1—C2	74.5 (2)	C3—C2—C11—C10	−0.1 (3)
C12—C1—C2—C11	−0.7 (2)	C1—C2—C11—C10	177.0 (2)
S—C1—C2—C11	174.62 (17)	C9—C10—C11—O1	174.94 (19)
C12—C1—C2—C3	175.5 (2)	C9—C10—C11—C2	−1.7 (3)
S—C1—C2—C3	−9.1 (4)	C2—C1—C12—O1	1.2 (2)
C11—C2—C3—C4	−177.8 (2)	S—C1—C12—O1	−174.72 (14)
C1—C2—C3—C4	6.3 (4)	C2—C1—C12—C13	−174.4 (2)
C11—C2—C3—C8	1.9 (3)	S—C1—C12—C13	9.8 (4)
C1—C2—C3—C8	−174.0 (2)	C11—O1—C12—C1	−1.1 (2)
C2—C3—C4—C5	−179.7 (2)	C11—O1—C12—C13	175.45 (17)
C8—C3—C4—C5	0.7 (3)	C1—C12—C13—C18	−146.4 (2)
C3—C4—C5—C6	0.0 (3)	O1—C12—C13—C18	38.2 (3)
C4—C5—C6—C7	0.2 (4)	C1—C12—C13—C14	38.3 (4)
C4—C5—C6—Br	−178.44 (17)	O1—C12—C13—C14	−137.1 (2)
C5—C6—C7—C8	−1.0 (4)	C18—C13—C14—C15	0.2 (3)
Br—C6—C7—C8	177.61 (16)	C12—C13—C14—C15	175.5 (2)
C6—C7—C8—C9	−177.6 (2)	C13—C14—C15—C16	0.0 (3)
C6—C7—C8—C3	1.6 (3)	C14—C15—C16—F	−177.9 (2)
C4—C3—C8—C7	−1.5 (3)	C14—C15—C16—C17	0.1 (4)
C2—C3—C8—C7	178.87 (19)	F—C16—C17—C18	177.6 (2)
C4—C3—C8—C9	177.7 (2)	C15—C16—C17—C18	−0.3 (4)
C2—C3—C8—C9	−2.0 (3)	C16—C17—C18—C13	0.5 (3)
C7—C8—C9—C10	179.4 (2)	C14—C13—C18—C17	−0.5 (3)
C3—C8—C9—C10	0.2 (3)	C12—C13—C18—C17	−175.9 (2)

Symmetry codes: (i) *x*+2, *y*, *z*−1.

### Hydrogen-bond geometry (Å, °)

**Table:**

Cg is the centroid of the C2/C3/C8/C9/C10/C11 ring.

**Table:**

*D*—H···*A*	*D*—H	H···*A*	*D*···*A*	*D*—H···*A*
C14—H14···O2A^ii^	0.93	2.53	3.235 (3)	133
C18—H18···Cg^iii^	0.93	2.65	3.347 (3)	132

Symmetry codes: (ii) *x*, −*y*+1/2, *z*−1/2; (iii) *x*+1, −*y*−1/2, *z*−1/2.
